# Identification of recurrent variants implicated in disease in bicuspid aortic valve patients through whole-exome sequencing

**DOI:** 10.1186/s40246-022-00405-z

**Published:** 2022-09-07

**Authors:** Shasha Chen, Qinchun Jin, Shiqiang Hou, Mingfei Li, Yuan Zhang, Lihua Guan, Wenzhi Pan, Junbo Ge, Daxin Zhou

**Affiliations:** 1 Department of Cardiology, Zhongshan Hospital, Fudan University, No. 180 of Road Fenglin, District Xuhui, Shanghai, 200032 China; 2 Research Unit of Cardiovascular Techniques and Devices, Chinese Academy of Medical Sciences, Shanghai, China; 3 National Clinical Research Center for Interventional Medicine, Shanghai, China

**Keywords:** Bicuspid aortic valve, Whole-exome sequencing, Aortic stenosis

## Abstract

Bicuspid aortic valve (BAV) is the most common congenital heart defect in human beings, with an estimated prevalence in the general population of between 0.5 and 2%. Moreover, BAV is the most common cause of aortic stenosis in the pediatric population. Patients with BAV may have no symptoms for life, and some of them may progress to aortic stenosis. Genetic factors increase the susceptibility and development of BAV. However, the pathogenesis and BAV are still unclear, and more genetic variants are still needed for elucidating the molecular mechanism and stratification of patients. The present study carried out screening of variants implicated in disease in BAV patients. The whole-exome sequencing (WES) was performed in 20 BAV patients and identified 40 different heterozygous missense mutations in 36 genes (*MIB2*, *FAAH*, *S100A1*, *RGS16*, *MAP3K19*, *NEB*, *TTN*, *TNS1*, *CAND2*, *CCK*, *KALRN*, *ATP10D*, *SLIT3*, *ROS1*, *FABP7*, *NUP205*, *IL11RA*, *NPR2*, *COL5A1*, *CUBN*, *JMJD1C*, *ANXA7*, *TRIM8*, *LGR4*, *TPCN2*, *APOA5*, *GPR84*, *LRP1*, *NCOR2*, *AKAP11*, *ESRRB*, *NGB*, *AKAP13*, *WWOX*, *KCNJ12*, *ARHGEF1*). The mutations in these genes were identified as recurrent variants implicated in disease by in silico prediction tool analysis. Nine genes (MIB2, S100A1, TTN, CCK, NUP205, LGR4, NCOR2, ESRRB, and WWOX) among the 36 genes were identified as variants implicated in disease via unanimous agreement of in silico prediction tool analysis and sequenced in an independent cohort of 137 BAV patients to validate the results of WES. BAV patients carrying these variants demonstrated reduced left ventricular ejection fractions (LVEF) (63.8 ± 7.5% vs. 58.4 ± 5.2%, *P* < 0.001) and larger calcification volume [(1129.3 ± 154) mm^3^ vs. (1261.8 ± 123) mm^3^, *P* < 0.001]. The variants in *TTN*, *NUP205* and *NCOR2* genes are significantly associated with reduced LVEF, and the variants in *S100A1*, *LGR4*, *ESRRB*, and *WWOX* genes are significantly associated with larger calcification volume. We identified a panel of recurrent variants implicated in disease in genes related to the pathogenesis of BAV. Our data speculate that these variants are promising markers for risk stratification of BAV patients with increased susceptibility to aortic stenosis.

## Background

Bicuspid aortic valve (BAV) is common congenital heart disease, and the prevalence rate is about 1 to 2% in the population. Some patients with BAV showed a family aggregation tendency [1]. Genetic studies showed that BAV had the characteristics of autosomal dominant inheritance and incomplete penetrance [2]. Aortic stenosis is the most common complication in patients with BAV. The pathophysiological basis of its formation includes endothelial dysfunction, local inflammation, lipid deposition, and secondary valve leaf emaciation [3]. Compared with patients with the tricuspid aortic valve, the time of valve stenosis in BAV patients is 10 years before, the progress is faster and higher mortality [4]. Once the patients with BAV have chest pain, syncope, and other symptoms, the alternative treatment is only valve replacement. However, there are some limitations and complications in mechanical or biological valves. The survival period of the untreated BAV patients with severe aortic stenosis is usually less than 10 years, especially for patients with heart failure [5]. Therefore, it is urgent to explore the pathogenesis of early calcification of BAV, carry out risk stratification for patients with asymptomatic BAV, delay the progression of the disease, and avoid surgery.

The heart valves of healthy people are composed of valve endothelial cells (VECs), valve interstitial cells (VICs), and extracellular matrix (ECM). VECs cover the valve surface, contact with blood, and maintain valve homeostasis by regulating permeability and inflammatory cell adhesion [6, 7]. VECs participate in heart valve formation through EndMT: endothelial to mesenchymal transformation [8]. VICs are the main cell groups of valve stroma, which constitute the skeleton of valve structure and play a role through their proliferation, differentiation, and secretion of ECM components. ECM provides physical and mechanical support for maintaining a certain morphological structure of the valve. The pathological characteristics of BAV are inflammatory infiltration, the synthesis of the fibrotic matrix after activation of valve interstitial cells (VICs), thickening, calcified mineral deposition in extracellular matrix (ECM), and then the obstruction of valve movement and blood flow. Calcification is a key process of aortic valve stenosis. When calcification occurs, alpha smooth muscle actin (*α*-SMA) can be activated and expressed in VICs, which can be transdifferentiated into myofibroblasts and show an osteoblast-like phenotype, which leads to massive calcium deposition and ossification, and eventually aortic valve stenosis [9].

Previous epidemiological studies have described the familial pattern of bicuspid aortic valve consistent with heredity and pointed out that genetic factors contribute more to disease susceptibility than environmental factors [10]. Genomic methods have just begun to elucidate the genetic determinants of BAV and have identified several pathogenic variants, such as *NOTCH1*, *GATA5*, *TGFBR1*, and *TGFBR2* [11]. However, the penetrance of BAV is low, and currently, reported genes are mostly a form of familial studies. On the other hand, BAV is a heterogeneous disease, and many unknown variations need to be identified in sporadic BAV patients.

The present study aims to find the possible characteristic mutation gene in BAV. Deployed a two-step strategy to evaluate the clinical significance of germline genetic markers in BAV patients. We carried out whole-exome sequencing (WES) in 20 BAV patients (WES cohort) to identify potential pathogenic genes by bioinformatics analysis and in silico prediction. Then we selected several candidate genes for sequencing in independent BAV patients (Validation cohort).

## Materials and methods

### Study population

Patients with bicuspid aortic valves were selected from the Department of Cardiology of our hospital from January 2018 to December 2020 and were diagnosed by transthoracic echocardiography. Inclusion criteria included: (1) age ≥ 18 years old; (2) echocardiographic results: Patients showed one or more punctate or annular echo enhancement of aortic valve with a diameter more than 1 mm.

Exclusion criteria included: (1) acute infection; (2) history of rheumatic disease; (3) infective endocarditis; (4) congenital aortic valve malformation, such as Marfan syndrome, Loeys-Dietz syndrome (LDS), and other congenital cardiac defects; (5) being treated with anti-osteoporosis drugs. Eventually, 157 BAV patients were collected in this study: 20 patients were part of the WES cohort for exon sequencing, and the other 137 were part of the validation cohort for Sanger sequencing on selected genes.

The validation cohort consisted of 137 BAV. We also collected 130 cases of physical examination in our hospital during the same period as the control cohort. They were all tricuspid aortic valves and excluded from heart valve disease by color Doppler echocardiography. The control cohort consisted of 76 males and 54 females with an average of 62.9 ± 10 years. This study was carried out by the principles of the Declaration of Helsinki and was approved by the ethics committee of Zhongshan Hospital. Informed consent was obtained from all patients.

### DNA extraction

Genomic DNA was isolated from peripheral whole blood samples that were cryopreserved under − 80 °C using QIAamp DNA Blood Mini Kit (Qiagen, Hilden, Germany) and was quantified using a fluorometer or a Microplate Reader (Qubit Fluorometer, Invitrogen, Carlsbad, CA), with 260/280 ratios ranging from 1.75 to 2.00 for all DNA samples. Agarose Gel Electrophoresis (concentration of agarose gel: 1%, voltage: 150 V, electrophoresis time: 40 min) detected sample integrity and purification.

### Whole‑exome sequencing

Genomic DNA extracted from whole blood samples was fragmented into 150 BP-220 BP by covaries, and the library was constructed and captured by Agilent sure select Human ALL Exon V6 kit. Terminal repair, Ploya tail addition, sequencing adaptor addition, purification, magnetic bead capture, PCR amplification, and other steps (Agilent Technologies, Santa Clara, CA, USA) finally constructed the DNA fragments. OEbiotech (Shanghai, China) performed next-generation sequencing on the Illumina HiSeq-2500 platform by BGI (Shenzhen, China). The average coverage of 190 × on target regions, of targeted bases, 99.91% was covered by at least 1 ×, and 99.34% was covered by at least 10 × coverage. Using BWA (Burrows-Wheeler Aligner) software, short reads mapping and alignment were performed. Single nucleotide polymorphisms (SNPs) were detected using GATK (Genome Analysis Tool Kit) v3.3.0 HaplotypeCaller. All reference sequences were based on the NCBI37/hg19 assembly of the human genome.

### Single nucleotide variant (SNV) analysis

We selected variants in exon or splicing sites. We only included nonsynonymous SNV, such as missense, nonsense, and splicing site with minor allele frequency (MAF) < 0.05 in both 1000Genome and 1000Genome East Asia databases. The potential impact of missense mutations on protein function was evaluated using SIFT and Polyphen, two computational methods. SIFT scores, ranging from 0 to 1. The SIFT score represents the probability of toleration for a particular amino acid substitution, ranging from 0 to 1, and a score below the cutoff value of 0.05 is generally considered harmful. Polyphen is used to calculate the posterior probability to predict the pathogenicity of mutation based on evolutionary conservatism and the protein's three-dimensional structure. The predicted results were D: potentially harmful (score = 0.957 ~ 1), P: possibly harmful (score = 0.453 ~ 0.956), B: benign (score = 0 ~ 0.452). The variants implicated in disease were assessed via in silico prediction tool analysis (SIFT and Polyphen). The recurrent pathogenic variant was defined as a variants implicated in disease that appeared at least in two patients in the WES cohort.

### Molecule annotation and network analysis

Single nucleotide polymorphisms (SNPs) were predicted and annotated by comparison using National Center for Biotechnology Information (NCBI) dbSNP version 141. Each SNP was mapped on the genome, and the number of SNP on detailed regions, such as coding region, untranslational region, an intron, was annotated. Nonsynonymous SNP information was extracted by comparing UCSC reference gene information (http://genome.Ucsc.edu/). Gene Ontology (GO) and KEGG pathway enrichment were analyzed by STRING online tools (http://string-db.org/).

### Statistical analysis

Quantitative variables were expressed as mean and standard deviation, and category variables were expressed as cases (percentage). Statistical analyses were carried out with Statistical Package for the Social Sciences (SPSS) 20.0. Continuous variables between two subgroups were compared using the unpaired two-sided* t*-test. Categorical variables were analyzed using Chi-square or Fisher’s exact tests. Patients whose data were missing were not included in the analysis. A* P*-value < 0.05 was considered statistically significant.

## Results

### General information of 20 BAV patients

WES analysis was performed on 20 BAV patients. There were 12 BAV male patients with an average age of 67 ± 12 years, among all had a mean aortic valve gradient ≥ 40 mmHg and aortic valve orifice area ≤ 0.8 mm^2^, and 3 (15%) had moderate or severe aortic valve regurgitation (Table 1).

**Table 1:** Baseline characteristics of 20 BAV patients

Variables	Summary statistics (*n* = 20)
*Patient characteristics*	
Male	60%
Age	67 ± 12
Arterial hypertension	40%
Diabetes mellitus	25%
Previous MI	0%
Hyperlipemia	30%
CKD (eGFR < 30 ml/min)	5%
COPD, moderate or severe	1%
STS risk score	2.7 ± 1.5
*Echocardiographic assessment*	
LVEF, %	66.7 ± 11.5
LVEDD, mm	59.2 ± 10.8
Mean aortic valve gradient ≥ 40 mmHg	100%
Aortic valve regurgitation, moderate or severe	15%
*CT scan*	
Aortic valve orifice area ≤ 0.8mm^2^	100%
Calcification volume (mm^3^)	1125.7 ± 268.3
*Mechanism of AS*	
Congenital bicuspid aortic valve	100%

### General features of whole-exon sequencing

WES analysis revealed an average of 299,980 SNPs (272,788 to 342,694) in 20 BAV samples. There are an average of 12,347 synonymous mutations in the overall SNP and 12,009 missense mutations in the coding region, including 108 SNPs making a stop codon and 15 SNPs making the stop codon a non-stop codon (Table 2).

**Table 2:** Characteristics of identified SNPs by individual samples

Muttype	Total	CDS	Synonymous	Missense	Stopgain	Stoploss	Intronic	Intergenic
BAV01	316,629	25,616	12,459	12,157	106	14	153,569	110,077
BAV02	316,788	25,060	12,272	11,774	117	13	155,191	109,148
BAV03	298,898	25,306	12,353	11,989	110	14	148,645	98,309
BAV04	281,407	25,049	12,149	11,959	125	12	142,253	88,484
BAV05	284,138	25,545	12,527	12,142	122	18	144,372	87,640
BAV06	288,281	25,062	12,333	11,950	103	16	142,722	94,138
BAV07	287,824	25,345	12,360	12,074	104	15	143,073	93,256
BAV08	319,790	25,367	12,348	11,965	109	13	157,454	109,216
BAV09	304,337	25,337	12,383	11,968	101	15	152,857	97,962
BAV10	306,704	25,673	12,415	12,272	98	14	152,682	101,295
BAV11	293,218	25,185	12,234	11,940	111	16	147,598	93,446
BAV12	298,719	25,430	12,454	12,058	109	19	146,054	101,176
BAV13	283,332	25,275	12,376	11,928	100	21	142,473	89,719
BAV14	272,788	24,997	12,274	11,881	106	12	138,540	83,700
BAV15	285,330	25,105	12,237	11,848	114	18	143,174	91,339
BAV16	322,148	25,097	12,275	11,886	108	12	157,732	111,678
BAV17	273,457	25,248	12,176	12,017	106	15	144,827	76,696
BAV18	292,524	25,540	12,501	12,071	108	16	147,862	92,285
BAV19	342,694	25,793	12,422	12,286	105	18	169,225	117,441
BAV20	330,585	25,388	12,399	12,017	104	12	174,702	98,433
Average	299,980	25,321	12,347	12,009	108	15	150,250	97,272

### Gene Ontology (GO) and KEGG pathway

We then filtered the results of the SNPs from sequencing to obtain the mutation gene, which changes the function of a protein. We compared the sequencing results of all samples to the reference genome, extracted all SNPs loci data for subsequent analysis, and obtained 37,225 SNPs loci. This SNPs site contains the site that changes the protein function and contains known high-frequency mutation sites. The synonymous mutation and unknown function mutation sites were removed. Then the SNPs were selected so that the variants have a MAF < 0.05 in both the 1000G and 1000G East Asia database. In the end, 14,862 SNPs sites from 9674 genes were left.

**Fig. 1:**
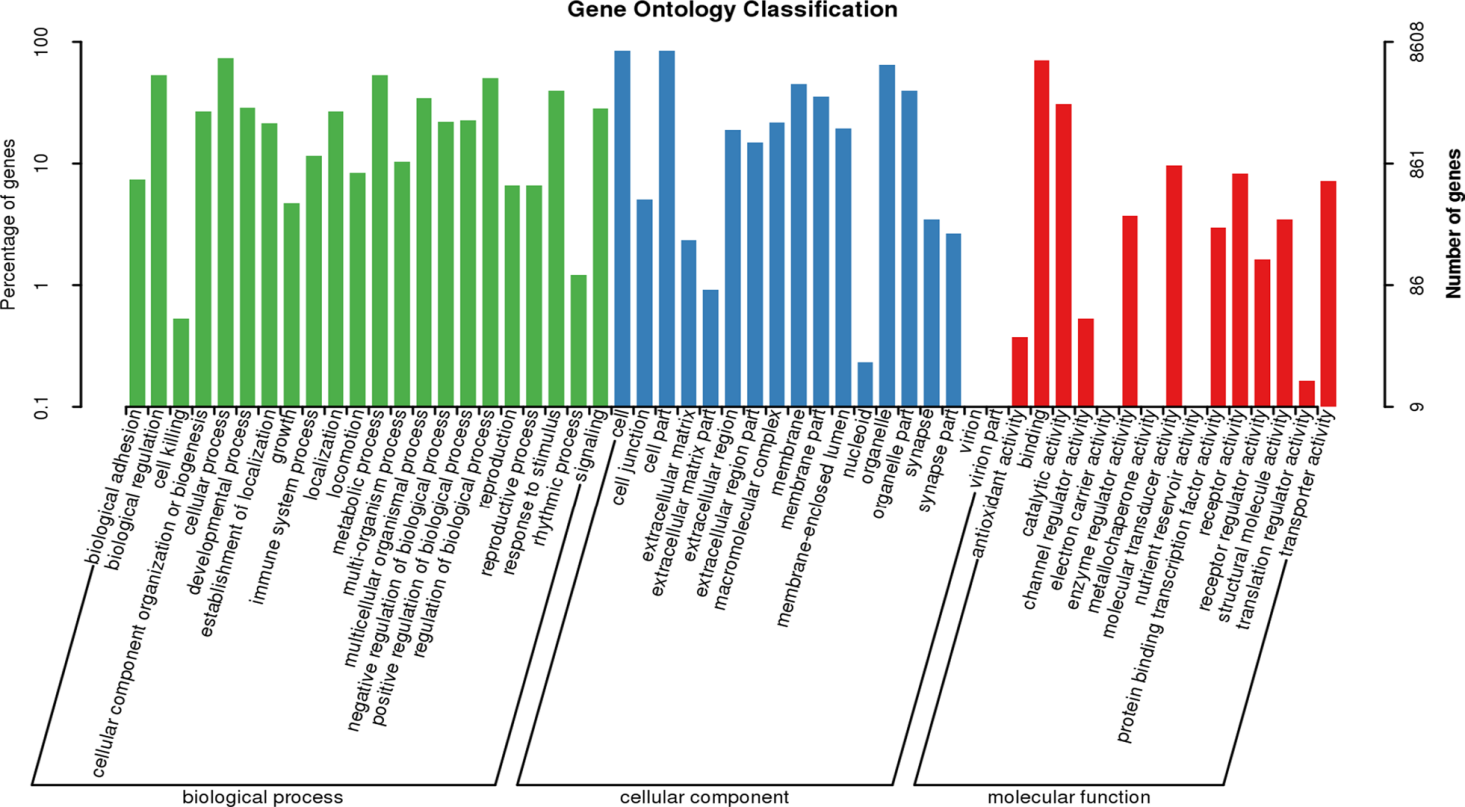
Gene Ontology of candidate genes with SNPs. This SNPs change the amino acid of protein and have a MAF < 0.05 in both 1000G and 1000G East Asia database. Only top enriched GO categories are shown in aspect of biological process, cell components and molecular functions from 9674 genes with 14,862 SNPs sites

**Fig. 2:**
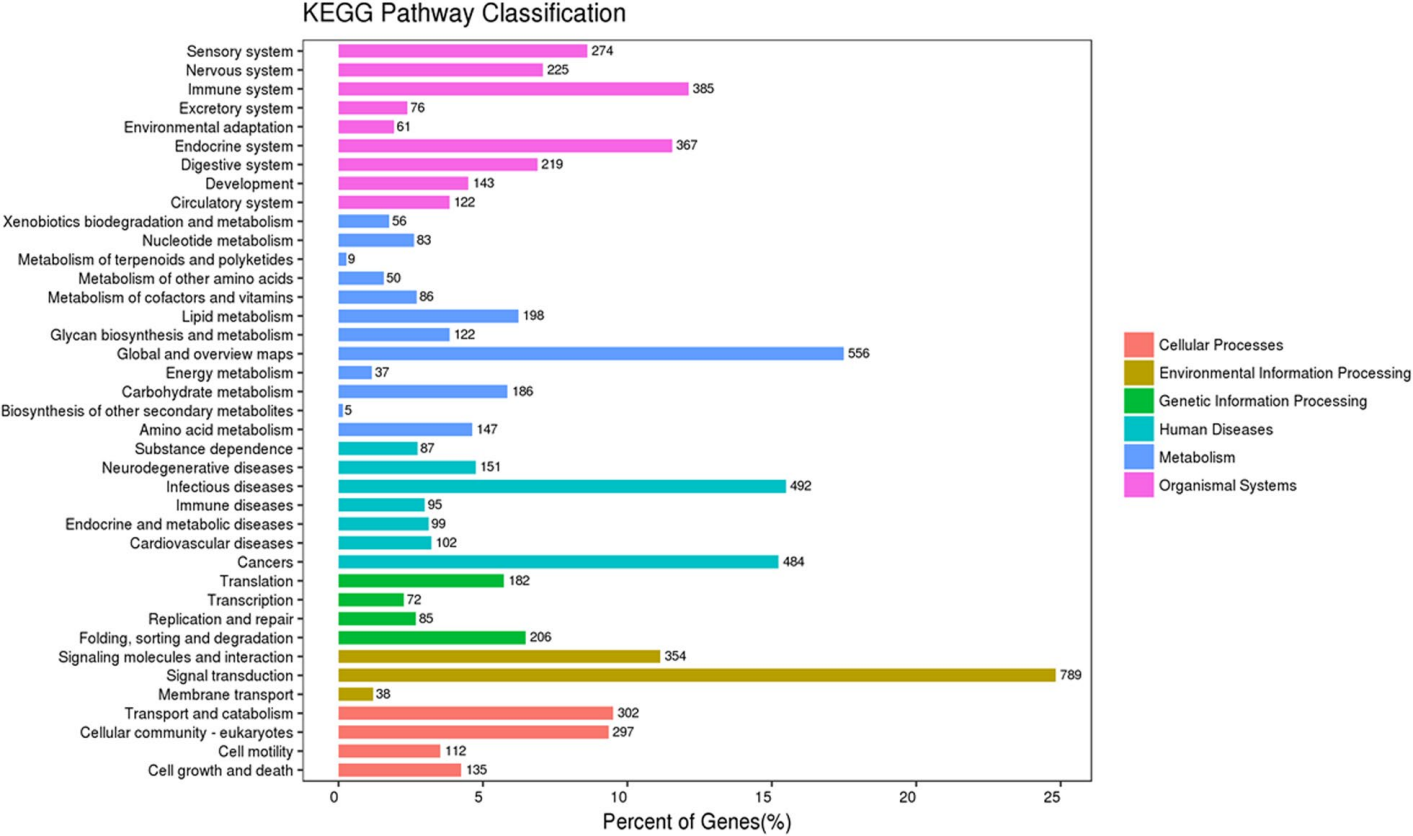
KEGG pathway analysis of candidate genes with SNPs. Only top enriched pathway categories were shown in aspect of cellular processes, environmental information processing, genetic information processing, human diseases, metabolism, and organismal systems

**Fig. 3:**
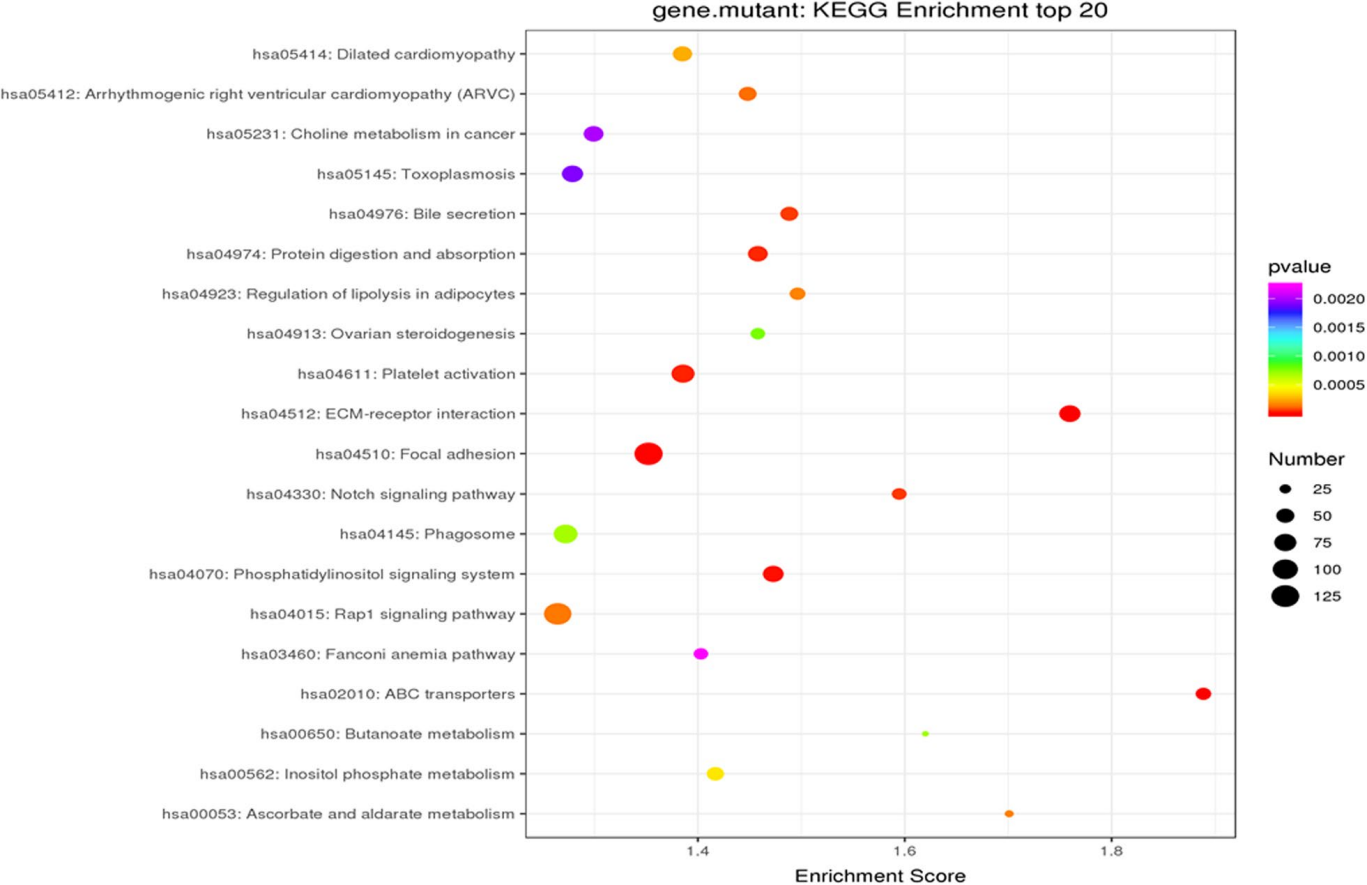
Enriched mutant genes by KEGG pathway analysis. Gene Ontology/pathway analysis of candidate genes. The top 20 pathways are shown

Gene Ontology (GO) and KEGG pathway enrichment were performed to analyze the most common molecular function and biological processes categories, respectively. Using the David database, 9674 genes were analyzed for GO. This analysis describes the three major components of the gene. The biological process is the main biological function of the gene-encoded protein; cell components are the main rich cellular areas of gene products; molecular functions are the possible activities of gene products at the molecular level. The top three enriched GO categories of SNP were cellular process, biological regulation, and metabolic process, cell part, organelle and membrane in cell components, binding, catalytic activity, and molecular transducer activity in molecular functions (Fig. 1). Based on the KEGG database and kobas database, signal pathway enrichment analysis was performed. And the top three enriched pathways of SNP were Signal transduction, Global and overview maps, and Infectious diseases (Fig. 2). Furthermore, the mutant gene's top three enriched KEGG pathways were Focal adhesion, Rap1 signaling pathway, and Phagosome (Fig. 3).

### Candidate recurrent variants implicated in disease in the WES cohort

The filtered variants were assessed via in silico prediction tool analysis (SIFT and Polyphen) to identify 1070 pathogenic variants. Two hundred forty-five recurrent variants were selected that appeared in at least two patients in the WES cohort [10]. Combined with a literature review for the biological function of genes with these variants, we identified 40 different recurrent pathogenic from 36 candidate genes, including *MIB2*, *FAAH*, *S100A1*, *RGS16*, *MAP3K19*, *NEB*, *TTN*, *TNS1*, *CAND2*, *CCK*, *KALRN*, *ATP10D*, *SLIT3*, *ROS1*, *FABP7*, *NUP205*, *IL11RA*, *NPR2*, *COL5A1*, *CUBN*, *JMJD1C*, *ANXA7*, *TRIM8*, *LGR4*, *TPCN2*, *APOA5*, *GPR84*, *LRP1*, *NCOR2*, *AKAP11*, *ESRRB*, *NGB*, *AKAP13*, *WWOX*, *KCNJ12*, *ARHGEF1* (Table 3).

**Table 3:** Identified recurrent variants implicated in disease in 20 BAV patients

Gene	dbSNP ID	Variant and AA change	Cases	1000G	1000G-EA
MIB2	rs376615315	c.C1153T:p.R385W	2	0.0002	0.001
FAAH	rs77101686	c.C1067T:p.A356V	2	0.008387	0.0159
S100A1	rs1046256	c.C261G:p.N87K	2	0.001597	0.0079
RGS16	rs191231364	c.T184G:p.W62G	2	0.000998	0.005
MAP3K19	rs56349597	c.G3122A:p.R1041H	2	0.003395	0.0169
NEB	rs139636644	c.C14183A:p.A4728D	2	0.011582	0.0417
NEB	rs149752325	c.G14182A:p.A4728T	2	0.011582	0.0417
TTN	rs56137800	c.C54886G:p.P18296A	2	0.004992	0.0248
TNS1	rs181295117	c.T2191A:p.S731T	2	0.000799	0.004
TNS1	rs181839905	c.C1500G:p.I500M	2	0.007987	0.0397
CAND2	rs180768267	c.A1847G:p.H616R	2	0.009784	0.0198
CCK	rs3774395	c.C283T:p.R95W,CCK	2	0.002596	0.0129
KALRN	rs78202770	c.C5084A:p.P1695Q	2	0.013578	0.0496
ATP10D	rs118048800	c.A221G:p.N74S	2	0.001198	0.006
SLIT3	rs2288792	c.G1184A:p.R395Q	2	0.004593	0.0228
ROS1	rs210968	c.T6720G:p.N2240K	2	0.038139	0.0248
FABP7	rs2279381	c.C182T:p.T61M	4	0.006989	0.0327
NUP205	rs145671518	c.C2356G:p.L786V	2	0.004393	0.0208
IL11RA	rs117149170	c.G782A:p.R261H	3	0.004193	0.0208
NPR2	rs114147262	c.C2368T:p.R790W	3	0.001597	0.0069
COL5A1	rs145178917	c.G378T:p.Q126H	2	0.007388	0.0347
CUBN	rs140806389	c.A6938T:p.Y2313F	2	0.009784	0.0486
CUBN	rs2271460	c.T6788G:p.F2263C	3	0.033746	0.0407
JMJD1C	rs117647164	c.A1253G:p.K418R	2	0.007388	0.0367
ANXA7	rs3750575	c.G1136A:p.R379Q	2	0.007788	0.0367
TRIM8	rs79218728	c.C718T:p.L240F	2	0.00639	0.0317
LGR4	rs149204548	c.G2176A:p.A726T	2	0.003195	0.0159
TPCN2	rs78034812	c.C2042T:p.S681L	5	0.010982	0.0387
APOA5	rs2075291	c.G553T:p.G185C	4	0.011382	0.0437
GPR84	rs77759698	c.T1108C:p.Y370H	3	0.006989	0.0347
GPR84	rs11170883	c.G110A:p.G37D	3	0.005791	0.0288
LRP1	rs79435985	c.A12161T:p.Y4054F	2	0.004792	0.0238
NCOR2	rs184942554	c.G3647A:p.R1216H	2	0.000599	0.001
AKAP11	rs2236364	c.C2162G:p.S721C	2	0.003794	0.0179
ESRRB	rs143477571	c.A79G:p.R27G	4	0.005391	0.0268
NGB	rs117207261	c.G178C:p.E60Q	3	0.000799	0.004
AKAP13	rs114777682	c.C568T:p.R190C	2	0.001797	0.005
WWOX	rs140817689	c.G129T:p.R43S	2	0.001198	0.006
KCNJ12	rs75029097	c.G433A:p.G145S	20	0.0002	0.001
ARHGEF1	rs2303797	c.C1025T:p.P342L	3	0.005791	0.0268

The variants are listed according to the chromosomal sequence (from 1 to X)

*BAV* bicuspid aortic valve, *TAV* tricuspid aortic valve, *dbSNP ID* single nucleotide polymorphism identification in database dbSNP

### Genetic markers in the validation cohort

**Fig. 4:**
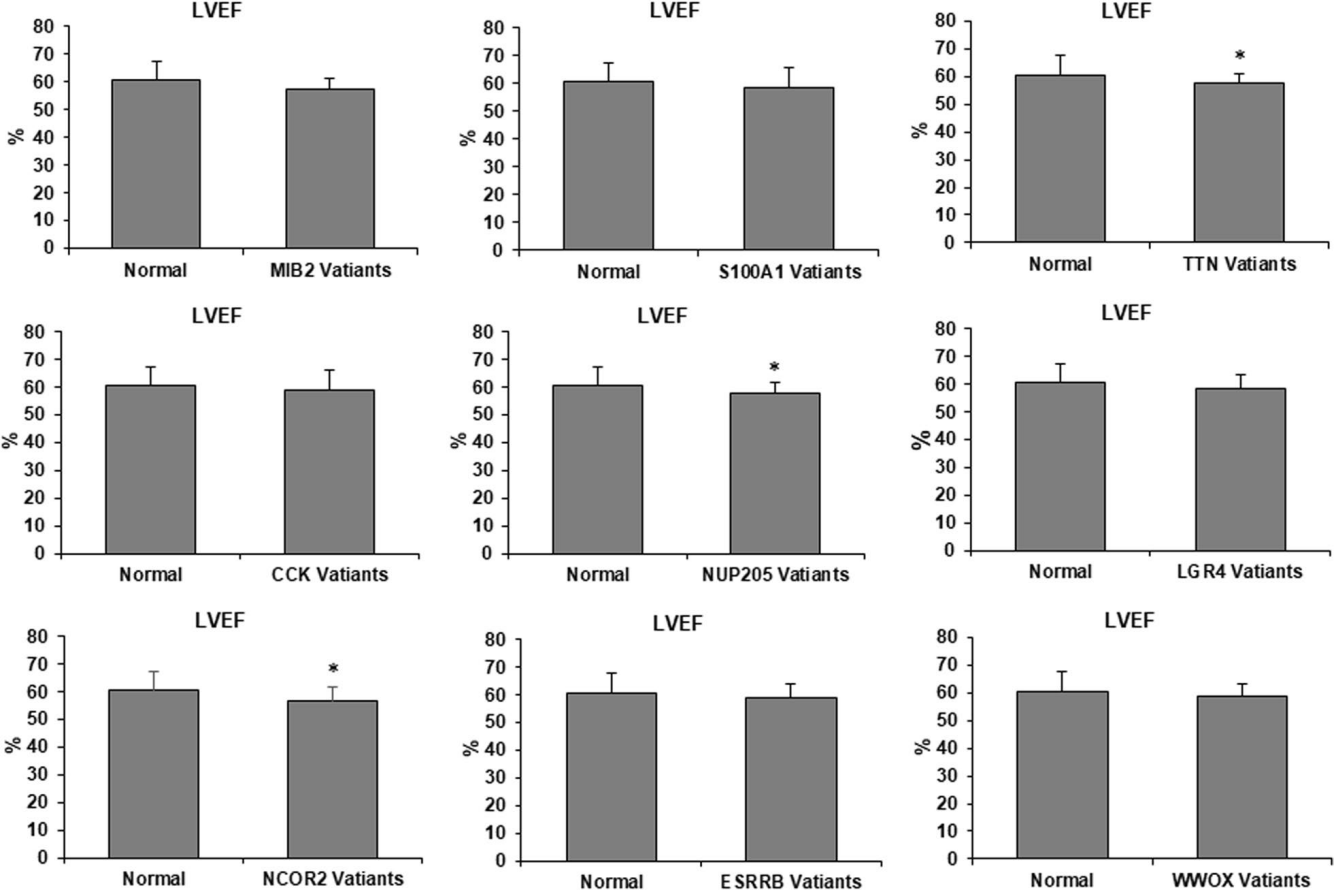
Comparison LVEF between normal and variant genes in BAV patients. Compared to patients with wide-type allele, patients with variant *TTN*, *NUP205* and *NCOR2* have significantly reduced LVEF. Values are expressed as Mean ± SD. *P < 0.05 versus wide-type group. LVEF, Left Ventricular Ejection Fractions

**Fig. 5:**
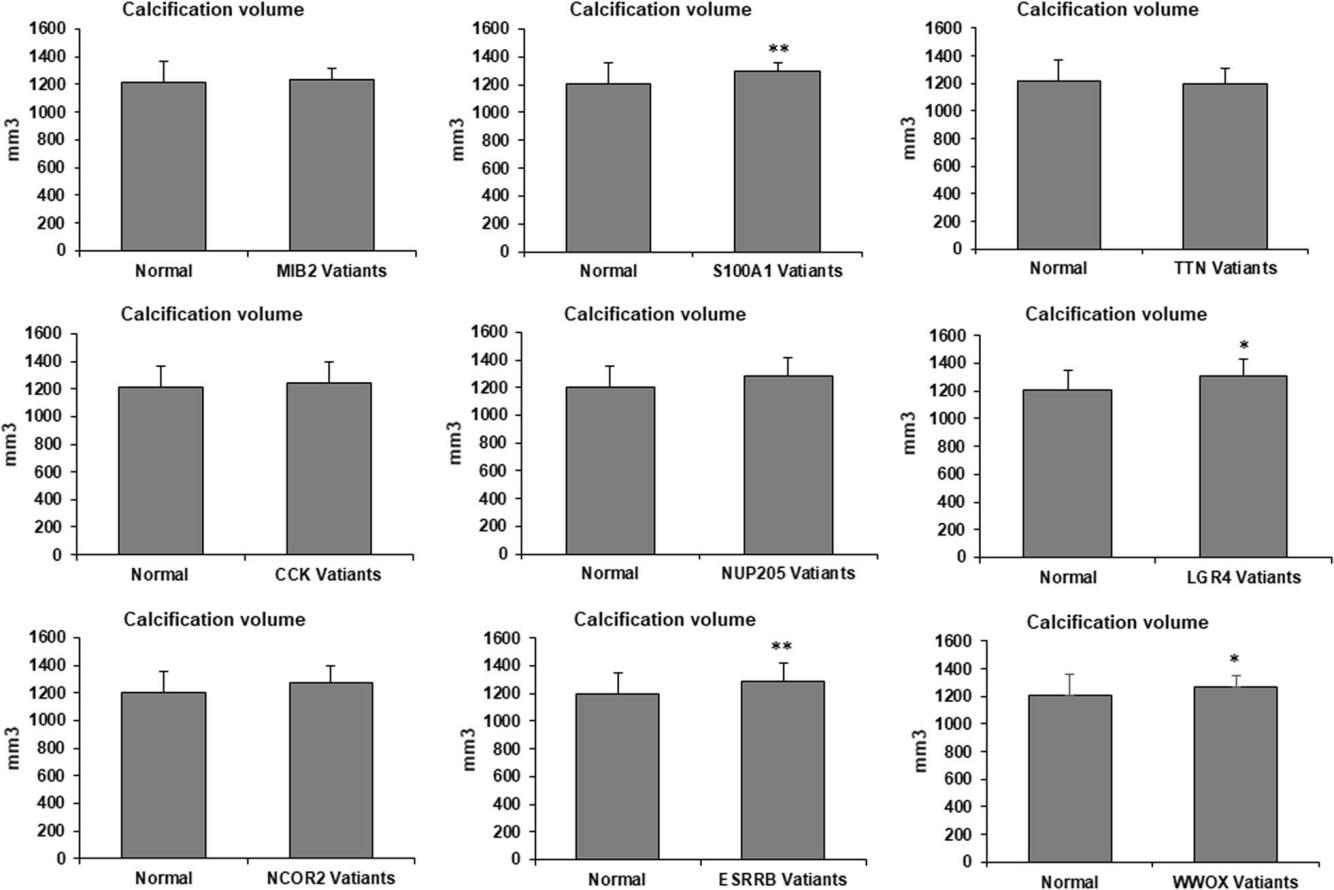
Comparison of calcification volume between normal and variant genes in BAV patients. Compared to patients with wide-type allele, patients with variant *S100A1*, *LGR4*, *ESRRB* and *WWOX* have significantly larger calcification volume. *P < 0.05, **P < 0.01 versus wide-type group

We performed a retrospective study on 137 BAV patients and sequenced their frozen DNA in 9 genes to confirm the WES results. These genes are chosen from 40 candidate genes with recurrent variants implicated in disease via unanimous agreement of in silico prediction tool analysis and are mostly related to BAV, including *MIB2*, *S100A1*, *TTN*, *CCK*, *NUP205*, *LGR4*, *NCOR2*, *ESRRB*, and *WWOX*. The panel of 9 variants implicated in disease was found in a total of 87 patients who had at least one heterozygous mutation among these genes, including 13 with *MIB2*, 11 with *S100A1*, 12 with *TTN*, 10 with *CCK*, 11 with *NUP205*, 14 with *LGR4*, 13 with *NCOR2*, 25 with *ESRRB*, and 14 with *WWOX*. The frequency of these 9 variants was significantly higher compared to healthy subjects with tricuspid aortic valves (Table 4). We then investigated the influence of these variants on the characteristics of BAV patients. Compared to 50 patients without a genetic marker, those harboring germline mutation demonstrated reduced LVEF, Left Ventricular Ejection Fractions (63.8 ± 7.5% vs. 58.4 ± 5.2%, *P* < 0.001), and larger calcification volume [(1129.3 ± 154) mm^3^ vs. (1261.8 ± 123) mm^3^, *P* < 0.001] (Table 5). We also divided all 137 BAV patients into wide-type and variant groups according to one of the nine genes to compare the LVEF and calcification volume. LVEF was significantly smaller in patients with variant *TTN*, *NUP205*, and *NCOR2* Compared to patients with wild-type alleles (Fig. 4). Furthermore, calcification volumes are significantly larger in patients with variant *S100A1*, *LGR4*, *ESRRB*, and *WWOX* than in patients with wide-type alleles (Fig. 5).

**Table 4:** The allele frequency of genetic markers identified in the validation cohort

Gene	Validation cohort *n* = 137	Control cohort *n* = 130	*P* value
MIB2	13	0	< 0.001
S100A1	11	1	0.004
TTN	12	3	0.020
CCK	10	2	0.022
NUP205	11	3	0.034
LGR4	14	2	0.003
NCOR2	13	0	< 0.001
ESRRB	25	4	< 0.001
WWOX	14	1	0.001

**Table 5:** Baseline characteristics of 137 BAV patients in the validation cohort

Variable	Validation cohort *n* = 137	Patients without mutation *n* = 50	Patients with mutation *n* = 87	*P* value
Gender	76 (55.4%)	29 (58%)	47 (54%)	0.652
Age	64.6 ± 10.8	64.4 ± 12.2	64.7 ± 10.1	0.862
Hypertension	50 (37.3%)	17 (34%)	33 (37.9%)	0.645
Diabetes	36 (26.3%)	11 (22%)	25 (28.7%)	0.389
Hyperlipemia	12 (8.8%)	29 (58%)	41 (47.1%)	0.251
LVEF (%)	60.4 ± 6.7	63.8 ± 7.5	58.4 ± 5.2	< 0.001
Calcification volume (mm^3^)	1213.4 ± 149.1	1129.3 ± 154	1261.8 ± 123	< 0.001

Data are presented as the mean ± SD, or as number (percentage)

## Discussion

In this study, we performed whole-exon sequencing on 20 sporadic BAV patients to explore the potential genetic variations that may contribute to the pathogenesis of BAV. We identified 40 different heterozygous missense mutations in 36 genes. These are recurrent variants implicated in disease in that they appeared in at least two patients and were selected by in silico prediction tool analysis from 14,826 nonsynonymous SNV in exons. Then nine genes (*MIB2*, *S100A1*, *TTN*, *CCK*, *NUP205*, *LGR4*, *NCOR2*, *ESRRB*, and *WWOX*) were selected for sequencing to validate the WES results in an independent cohort of 137 BAV patients. 87 patients carry at least one variant, and 50 patients do not have any variant among these nine genes. Patients with germline mutations showed reduced LVEF and larger calcification volume than patients with a wide-type allele in all nine genes. The data indicate that these genes with recurrent variants implicated in disease may involve the pathogenesis of BAV.

This study speculated the hypothesis that genetic variations increase the susceptibility to BAV. Here we rediscovered two genes, *COL5A1* and *KCNJ12*, in BAV patients. *COL5A1* is an ECM-related genes, and its variant (*COL5A1*: c.A3481T:p.I1161F) was identified as variants implicated in disease in BAV [12]. We identified a new variant of *COL5A1* (c.G378T:p.Q126H; rs145178917) in BAV, a common SNP (1000G-EA: 0.0347). Another reported gene is *KCNJ2*, and its heterozygous missense mutation (*R67W*) was detected in Andersen syndrome with cardiovascular malformation of the bicuspid aortic valve [13]. Interestingly, a heterozygous mutation in *KCNJ12* (p.Glu334del) was identified as a candidate mutation in dilated cardiomyopathy [14], whose mutation site is close to our results (p.G145S). Whether *KCNJ12* plays a role in the pathological mechanism of BAV remains unclear.

Due to the low penetrance and heterogeneity of BAV, many unknown genes may influence the susceptibility and progression of BAV, especially in sporadic BAV. This study uncovered many variants of candidate genes that have not previously been implicated in BAV. These genes that carry recurrent variants implicated in disease can be divided into several main cellular and molecular mechanisms associated with BAV. Some mutated genes are related to atherosclerosis, such as *FAAH* [15], *KALRN* [16], *ATP10D* [17], *CUBN* [18], *APOA5* [19], and *LRP1* [20]. Atherosclerosis share several molecular mechanisms with BAV, including dyslipidemia and the activation of specific pro-inflammatory pathways (*NLRP3* inflammasome and *TRL4*) [21]. The SNPs in *KALRN* (rs9289231), *ATP10D* (rs2351791), *CUBN* (rs2291521), and *APOA5* (Rs662799) are all significantly associated with the risk of coronary artery disease (CAD). Cardiac hypertrophy is common in BAV patients with increased LV mass and reduced aortic elasticity [22]. Genes associate with cardiac hypertrophy included *JMJD1C* [23], *ANXA7* [24], *TRIM8* [25], *NGB* [26], and *AKAP13* [27]. Cardiac fibrosis is another pathogenic process in BAV. BAV patients with left ventricular (LV) fibrosis were more likely to progress to aortic stenosis that needed aortic valve replacement [28]. We also detected variations in genes involving cardiac fibrosis, such as *CCK* [29], *SLIT3* [30], *IL11RA* [31], and *ARHGEF1* [32]. Some identified genes are involved in the osteogenesis process, including *GPR84* [33] and *AKAP11* [34]. Other candidate genes are a pathway of known genes in BAV. For instance, *MAP3K19* is a regulator of TGF-*β* [35], and *FABP7* is a target of Notch1 [36].

We also sequenced 9 recurrent pathogenic genes for validation, whose allele frequency was significantly higher than healthy subjects with the tricuspid aortic valve. Patients with variant *TTN*, *NUP205*, and *NCOR2* had significantly smaller LVEF than patients with wide-type alleles. The finding indicates the mutations *TTN*, *NUP205*, and *NCOR2* can enhance the severity of aortic valve stenosis, a consequence of BAV. *TTN* gene encodes Titin, and it is a giant sarcomeric protein that regulates passive myocardial stiffness. The expression of less Titin isoform (N2BA and N2B) was changed in left ventricular biopsies of patients with aortic stenosis [37]. This change in Titin is in response to pressure overload and might further promote myocardial fibrosis or severe aortic stenosis [38]. *NUP205* can modulate cilia function, and its depletion leads to loss of cilia and abnormal cardiac morphology [39]. Cilia participate in aortic valve morphogenesis, and recently defects in the cilia machinery have been discovered as a causal factor in BAV and aortic stenosis [40, 41]. *NCOR2* is related to the Notch signaling pathway [42], but its role in BAV is unclear.

We found 4 genes, including *S100A1*, *LGR4*, *ESRRB*, and *WWOX*, are associated with the calcification volume of BAV patients. *S100A1* modulates the molecular pathways and signaling cascades in cardiomyocytes, endothelial cells, and cardiac fibroblasts [43]. It modulates the function of cardiomyocytes via TLR4/ROS/NF-κB pathway [44], which is involved in enhanced osteogenic responses in human aortic valve cells [45]. *LGR4* protects against ischemic injury of cardiomyocytes by modulating mitochondrial function and oxidative stress [46]. *GPR48* also is another receptor for *RANKL* modulating osteoclast differentiation [47]. *ESRRB* can decrease calcium sensitivity in cardiomyocytes and thus promote cardiomyocyte contractility [48]. *WWOX* can modulate cellular lipid homeostasis by increasing serum HDL cholesterol concentrations, which may affect the progression of atherosclerotic disease [49]. Genome-wide association study of the gene showed genetic variants in *WWOX* are correlated with coronary artery calcification [50].

## Limitation

The current investigation did not provide any additional evidence that detected genetic variants were responsible for the clinical manifestations of BAV patients. The present study's limitations were surmounted by the in vitro confirmation of these variations' biological effects, which warranted further investigations. To see a complete picture of the variant interpretation, more recent prediction tools (e.g., CADD) and a more recent genome-scale database (e.g., gnomAD) could be used.

## Conclusion

**Fig. 6:**
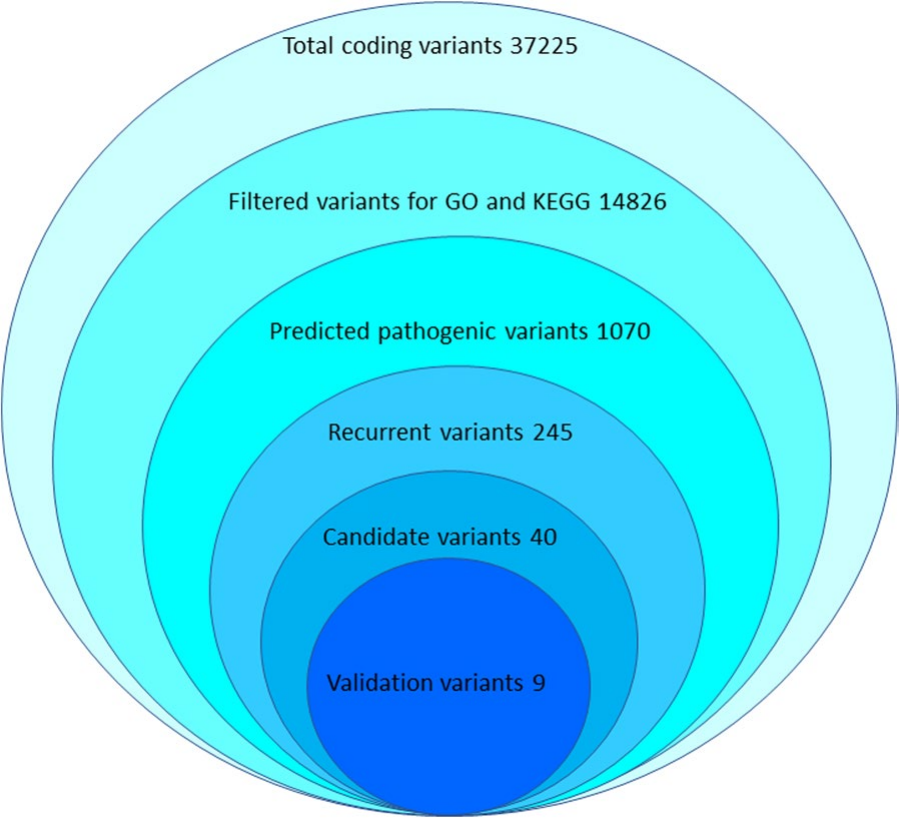
The selection process for WES data. It contains 37,225 total coding variants with nonsynonymous mutation. Then filtered 14,826 common variants for GO and KEGG analysis. One thousand and seventy variants implicated in disease are selected via in silico prediction tool analysis, 245 recurrent variants implicated in disease are selected that exist in at least two patients. Forty candidate variants among 36 genes are selected after the literature review that may be associated with phenotype of BAV. Finally, 9 genes were selected for validation in another cohort of 137 BAV patients by sequencing

In sum, we performed whole-exon sequencing in 20 sporadic BAV patients. We found 40 recurrent variants implicated in disease in 36 genes, and 9 variants were validated in another cohort of BAV patients (Fig. 6). Recurrent missense mutations on *TTN*, *NUP205*, *NCOR2*, *S100A1*, *LGR4*, *ESRRB*, and *WWOX* could be identified as potential pathogenic genes and associated with an elevated allele frequency, reduced left ventricular ejection fractions, and larger calcification volume in BAV patients.

## Data Availability

The data reported in this study are available upon a valid request from the corresponding author.
